# Influence of Thermal Aging on Space Charge Characteristics and Electrical Conduction Behavior of Cross-Linked Polyethylene Cable Insulation

**DOI:** 10.3390/polym16111600

**Published:** 2024-06-05

**Authors:** Jie Yang, Ruizhe Li, Leiyu Hu, Weiwang Wang

**Affiliations:** 1 Engineering Training Center of CAVTC, Chengdu Aeronautic Polytechnic, Chengdu 610100, China; yjandz@163.com; 2 State Key Laboratory of Electrical Insulation and Power Equipment, Department of Electrical Engineering, Xi’an Jiaotong University, Xi’an 710049, China; lrz6697056@stu.xjtu.edu.cn (R.L.); leiyuhu@stu.xjtu.edu.cn (L.H.)

**Keywords:** cross-linked polyethylene, thermal ageing, space charge, conduction

## Abstract

The aging of cable insulation presents a significant threat to the safe operation of cables, with space charge serving as a crucial factor influencing cable insulation degradation. However, the characteristics related to space charge and conduction current behavior during thermal aging remain unclear. This study focused on the thermal aging of cross-linked polyethylene (XLPE) material and utilizes a combined pulse electro-acoustic (PEA) and conduction current testing system to analyze the space charge and conduction current characteristics in the sample under varying electric fields and temperatures. The average charge density, short-circuit residual electric field, electric field distortion rate, and conduction current were studied. The findings indicate that the space charge in the samples following thermal aging is predominantly governed by the injected charge. The amorphous region of XLPE decreases, while the cross-linking degree increases after aging, thereby facilitating charge carrier migration within the sample and reducing the generation of charge carriers through thermal pyrolysis. The minimum temperature required for charge injection is reduced by thermal aging. Furthermore, modifications in conduction current, residual electric field, and average charge density indicate that thermal aging has the potential to alter the microstructure and trap characteristics of XLPE. This study provides empirical evidence to elucidate the underlying mechanism of cable insulation aging.

## 1. Introduction

High-voltage direct current (HVDC) cables play a crucial role in power transmission engineering [1]. XLPE is extensively used as the insulation material for HVDC cables due to its exceptional electrical, thermal, and mechanical properties [2,3]. However, during cable operation, factors such as electricity, heat, mechanical stress, and moisture can induce thermal aging [4], partial discharge [5], electrical tree aging [6], and water tree aging [7]. Recent research has demonstrated that the pyrolysis reaction of molecular chains during thermal aging leads to an increase in the carbonyl index of materials [8], a decrease in crystallinity [9], and a reduction in activation energy [10]. Consequently, significant alterations occur in the physical and chemical structure of XLPE cable insulation, which can ultimately result in insulation failure [11].

The aging of cables is closely associated with the injection and accumulation of charge carriers [12,13]. Three related aging mechanism models have been proposed: the DMM (Disado LA, Mazzanti G, and Montanari G) space charge model [14], the kinetic model suggested by Lewis et al. [15], and the thermodynamic model proposed by Crine et al. [16]. According to the DMM space charge model, the buildup of space charges induces electric field distortion within the polymer, resulting in aging and breakdown. The Lewis model posits that electrically induced mechanical stresses play a significant role in contributing to electrical breakdown. The Crine model suggests that robust charge injection occurs only subsequent to nano-cavity formation, wherein space charge is an outcome of aging. Currently, research on the aging of XLPE cables primarily focuses on two aspects: firstly, investigating the physicochemical, dielectric, and mechanical properties of XLPE insulation after aging; FTIR analysis revealed an increased absorption peak of carbonyl groups in thermally aged XLPE, which correlated with prolonged thermal aging time [17]. Secondly, exploring the space charge characteristics of XLPE. Huang et al. observed a gradual increase in dynamic charge ratio, dielectric constant, and conductivity of XLPE insulation with extended thermal aging time [18]. L. A. Disado et al. conducted multiple stress life tests on flat specimens of polyethylene terephthalate (PET) at various temperatures and electrical stress levels. They performed a quantitative analysis to investigate the relationship between space charge and the electrothermal lifetime of PET, and proposed a novel thermoelectric model [19]. However, the relationship between space charge accumulation, conduction current behavior, and the residual electric field during thermal aging remains unclear. In terms of investigating the aging mechanism of XLPE and its detection, Miceli et al. conducted numerical simulations to explore the mechanisms through which micrometric pores within XLPE can expand and merge (known as water treeing) [20]. Zhang et al. evaluated the applicability of reflectometry techniques for monitoring insulator damage in electric cables [21].

The focus of this study was to investigate the characteristics of space charge and conduction current exhibited by thermally aged XLPE cable insulation. This article conducts a measurement study on the distribution of space charge and conductivity current following aging of cross-linked polyethylene, investigating the alterations in space charge, electric field, and conductivity of cross-linked polyethylene after accelerated thermal aging. Additionally, it examined the variations in space charge with respect to electric field, temperature, and degree of thermal aging based on existing research. The combined PEA and conduction current testing system was employed to investigate the influence of electric field strength and temperature on the space charge distribution and electric field distortion characteristics of XLPE insulation with varying aging times. Furthermore, an analysis was conducted of the electrical conduction characteristics of thermally aged XLPE samples, elucidating the mechanism behind the impact of thermal aging on both space charge behavior and electrical conduction in XLPE. These research findings provide experimental and theoretical support for comprehending the relationship between thermal aging of XLPE insulation materials and their associated space charge phenomena, as well as electrical conduction.

## 2. Materials and Methods

### 2.1. Sample Preparation

The low-density polyethylene (LDPE) used in this study was obtained from DuPont Corporation, with a particle size of 4.5 mm and a density of 0.922 g/cm^3^. XLPE samples were prepared as follows: initially, the LDPE particles were melted at 125 °C and 5 MPa; subsequently, an appropriate amount of LDPE was uniformly placed on the mold; then, it underwent plate-vulcanization under conditions of 180 °C and 15 MPa for a duration of 10 min; finally, the samples were cooled down to room temperature under a pressure of 15 MPa. The resulting samples were cut into round shapes with a diameter of 5 cm and washed with anhydrous ethanol. Subsequently, the washed samples were dried in a vacuum drying oven at 125 °C for a period of 12 h.

### 2.2. Thermal Aging Experimental Setup

The XLPE samples were divided into nine groups and subjected to thermal aging experiments in a DKN412C constant temperature drying oven. The cross-linked polyethylene exhibits a maximum operating temperature of 90 °C, with a softening temperature of 130 °C. Therefore, an intermediate aging temperature of 100 °C was selected. To ensure uniform heating of each sample, the spacing between samples inside the oven was maintained at a minimum of 10 cm. Furthermore, to address issues related to specimen softening and bonding caused by heating, we placed the specimens on a dust-free cloth made of Teflon material. During the aging process, a subset of samples was periodically extracted from the oven every 48 h for performance testing. Table 1 shows the information of prepared XLPE unaged and aged samples.

### 2.3. Characterization of Materials

**Figure 1:**
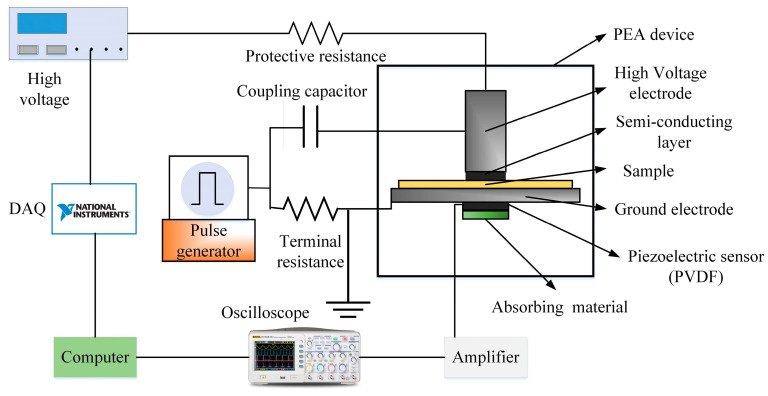
PEA space charge measurement system.

A typical PEA space charge test platform is illustrated in Figure 1. Previous studies have elucidated the configuration of the PEA system [22,23]. The electric stress is imparted by a high-voltage (HV) amplifier, which is connected to a high-voltage electrode via a series resistance. To ensure acoustic impedance matching between the HV electrode and the sample, a semiconductor (SC) layer was employed. Pressure waves with an amplitude of 500 V (at 1 kHz) and pulse width of 5 ns were generated using a pulse generator. A PVDF film served as the piezoelectric transducer for detecting the output signal. 

In the experiment, a joint measurement method combining conductivity current and space charge was employed to investigate XLPE film. The sample was rapidly switched between different environmental temperatures and external field strengths to examine the distribution of both external current and space charge. By analyzing the spatial distribution of space charge, the electric field distribution can be determined, allowing for calculation of the apparent mobility of free charge carriers through decay rate analysis. Consequently, the displacement current density within the sample was evaluated, enabling deduction of its component from the external current density in order to obtain a comprehensive spatiotemporal profile of conductivity current density.

## 3. Results and Discussions

### 3.1. DSC and SEM Analysis

**Figure 2:**
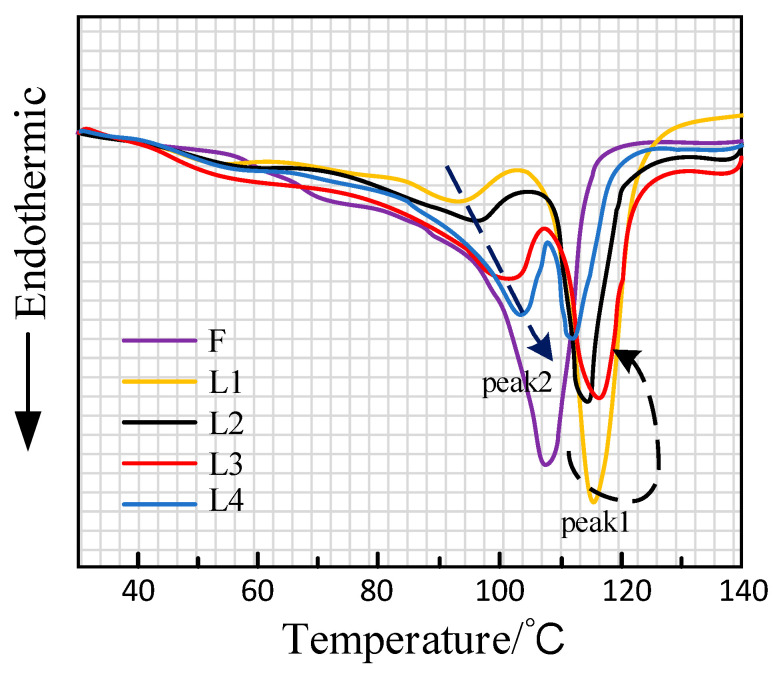
DSC curves of unaged and thermal aged XLPE samples.

The DSC method was used to analyze the melting characteristics of crystals in thermally aged XLPE samples. Figure 2 shows the DSC curves of different thermal aging samples. It can be observed that the unaged sample’s DSC curve only exhibits one crystal melting peak (peak 1) around 108 °C. It indicates that when the aging temperature is set at 100 °C, only a portion of imperfect crystals of the unaged sample is melted.

The crystalline melting peak of XLPE aged samples exhibited a phenomenon of multiple peaks. Apart from peak 1, a small melting peak, peak 2, is observed around 100 °C. As the aging time increases, peak 2 shifts towards a higher temperature and its area gradually increases. Considering the thermal process of XLPE samples during aging, peak 2 represents the DSC thermal history peak. On the other hand, peak 1 initially moves to a higher temperature and its region increases with aging time but later shifts towards lower temperatures and its area decreases.

**Figure 3:**
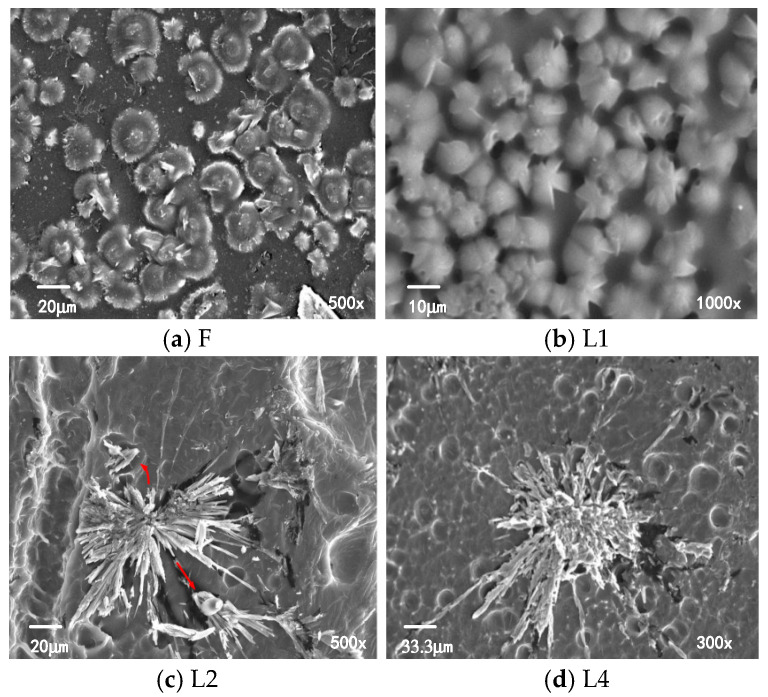
SEM results of unaged and aged XLPE samples.

Figure 3 shows the SEM results of XLPE samples. The spherulites of unaged XLPE samples are formed by tightly packed regular single crystals, with a diameter of approximately 10 to 20 μm. The regions between the spherulites are amorphous areas. The spherulite structure of sample L4 remains largely intact, but some spherulites show “petal-like” defects, as shown in Figure 3b. The diameter of the spherulites in sample L4 is approximately 10 to 15 μm. After serious aging, the number of lamellae comprising the spherulites decreases, and the spacing between lamellae increases, leading to the disruption of the integrity of the spherulite structure. The diameter of spherulites significantly increases, reaching approximately 50 μm, as shown in Figure 3c,d.

The three-dimensional network structure formed by cross-linking reaction inhibits the formation of large-sized spherulites in XLPE and promotes an increase in the number of spherulites. In aged samples, the antioxidants are depleted compared to in unaged samples, resulting in a decrease in cross-linking density. Due to the absence of antioxidants and decreased cross-linking density, fewer spherulites are formed in aged samples, while the size of spherulites is larger than that in the unaged samples.

### 3.2. Space Charge Distribution Characteristics

To investigate the impact of the electric field on the spatial distribution of space charge in XLPE samples, various DC voltages with different electric field magnitudes were applied to both unaged and aged specimens. The electric field strength ranged from 20 kV/mm to 80 kV/mm at intervals of 20 kV/mm, while the polarization duration was set at 1800 s followed by a short-circuit for 600 s. Data acquisition was performed every 10 s using a computer system to record the spatial distribution of space charge and external current within the sample.

**Figure 4:**
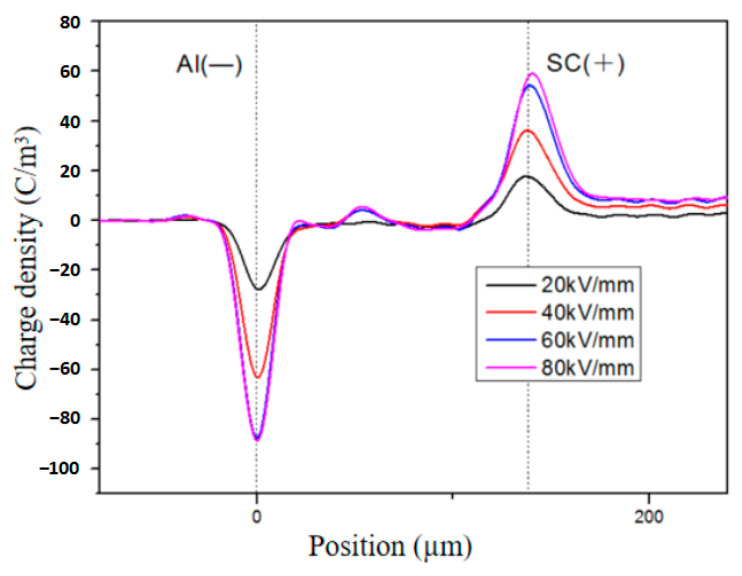
Space charge distribution of unaged XLPE under different electric fields.

The space charge distribution of the unaged XLPE under different electric fields is illustrated in Figure 4. It can be observed that there is a buildup of homocharges near the electrode and positive charges within the sample. Under an electric field strength of 20 kV/mm, the space charge distribution exhibits minimal fluctuations, indicating negligible presence of space charge within the sample. However, at 40 kV/mm, a small quantity of homocharges accumulates near the anode with a charge density ranging from 5 to 10 C/m^3^. As the electric field strength increases to 60 and 80 kV/mm, there is a significant increase in homocharge accumulation near the anode compared to lower electric fields, accompanied by a small number of homocharges appearing near the cathode. These findings suggest that under low electric fields, there is virtually no charge present inside the unaged sample. Nevertheless, when subjected to an electric field intensity of 40 kV/mm, reduced injection barriers facilitate easier movement of electrons and holes across these barriers into the interior region [24]. Consequently, charges injected by electrodes are captured near their contact interface with samples leading to homocharge accumulation. With further escalation in applied electric field strength, both holes and electrons injected from electrodes migrate towards cathodes and anodes, respectively, due to electrical stress forces resulting in the appearance of positive charges within samples.

**Figure 5:**
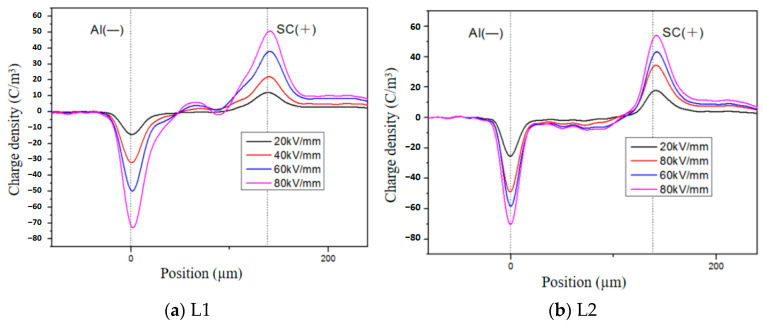

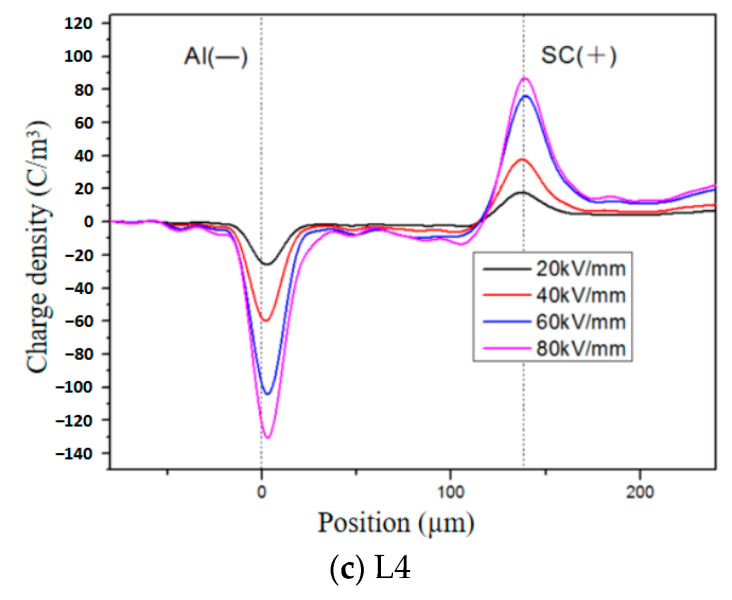
Space charge distribution of XLPE under different electric fields after thermal aging.

Taking XLPE samples aged for 4, 10, and 14 days as examples, this study analyzed the influence of electric field strength on the spatial distribution of space charges within the samples. Figure 5a presents the test results of space charge distribution in a sample aged for 4 days. It can be observed that at electric field strengths of 40 and 60 kV/mm, the injected charge density ranges from approximately 5 to 10 C/m^3^. With an increase in electric field to 80 kV/mm, the injected charge density further rises to around 15–20 C/m^3^. Notably, positive charge accumulation is also observed within the sample after aging for four days when compared with unaged samples. This phenomenon may be attributed to both migration of charges injected from electrodes and movement of charge carriers generated by molecular pyrolysis towards electrodes under electric field stress [25], resulting in space charge accumulation within the sample. Moreover, at lower applied electric fields, accumulated charges within the sample are primarily derived from pyrolysis processes. As the applied electric field increases further, a reduced Schottky injection barrier enhances injected charge amounts and gradually dominates within the sample.

The space charge distribution under different electric fields for samples aged for 10 days and 14 days is shown in Figure 5b,c. It can be observed that compared to aging for 4 days, the accumulation of homocharges near the anode decreases in the sample aged for 10 days, resulting in a disappearance of positive charges within the sample. Furthermore, in the case of the sample aged for 14 days, there is an accumulation of homocharges near the cathode and negative charges accumulate within the sample. Consequently, pyrolysis generates fewer charge carriers and leads to a decrease in positive charges within the sample. Additionally, aging-induced reduction in amorphous regions in XLPE promotes migration of charge carriers within the sample [26]. Therefore, long-term aging results in a decrease in homocharges near the anode due to recombination effects caused by electrons injected from the cathode; however, unrecombined electrons accumulate within the sample forming negative space charge accumulations. 

**Figure 6:**
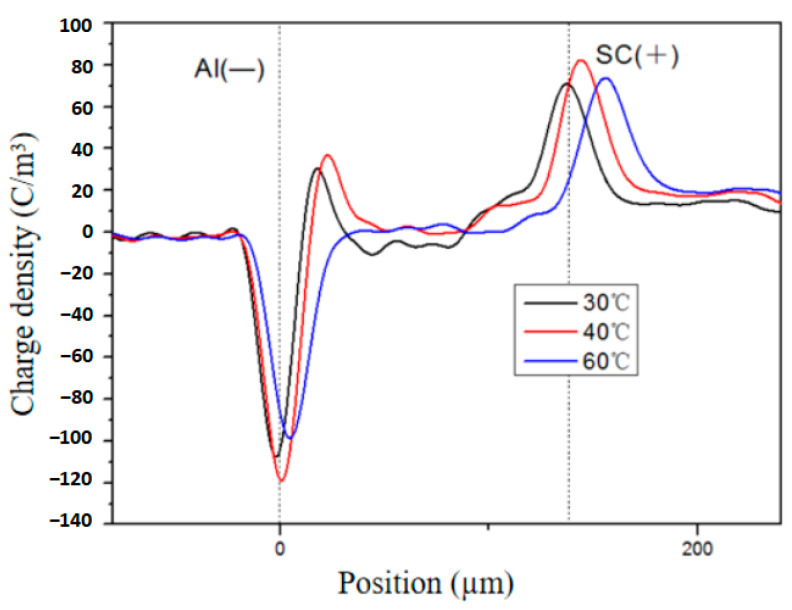
Space charge distribution of XLPE aged for 8 days at different temperatures.

The temperature has always been a crucial factor influencing the space charge behavior of polymer dielectrics [27,28]. As an illustration, Figure 6 demonstrates the impact of temperature on the spatial distribution of space charge in a sample aged for 8 days. It is evident that heterocharges are observed near the cathode, while homocharges are injected from the electrode. At lower temperatures, an increase in temperature leads to a higher accumulation of heteropolar space charges. However, as the temperature continues to rise, the extent of heteropolar charge accumulation diminishes. At 30 °C, a significant accumulation of heterocharges near the cathode is observed, with a charge density ranging from 20 to 40 C/m^3^. The charge distribution curve of the sample exhibits minimal fluctuations, indicating limited injection of space charges from the electrode at 30 °C. At 40 °C, there is still an accumulation of heterocharges near the cathode, with a higher charge density compared to that at 30 °C. Additionally, notable positive charges appear within the sample. Upon reaching a temperature of 60 °C, there is a sharp decrease in heterocharges near the cathode while a small quantity of homocharges is present near the anode. Comparative analysis reveals that as the temperature increases from 30 °C to 40 °C, more heterocharges accumulate at the cathode due to enhanced pyrolysis reaction and increased generation of heterocharges. When the temperature rises to 60 °C, the reduced potential barrier for charge passage through interfaces enhances the recombination effect, leading to a decreased presence of heterocharges and a gradual increase in homocharge accumulation near electrodes. Notably, as temperature increases, there is significant shifting observed in the space charge peak waveform due to the slower propagation speed of sound waves within electrodes, resulting in prolonged signal propagation time [29].

### 3.3. Space Charge Accumulation and Dissipation Characteristics of Thermally Aged XLPE

The amount of space charge and the average charge density can be calculated by analyzing the spatial distribution of charges within the sample [30,31]:

(1) $$Q(t)=\int_{0}^{d}\left|\rho (x,t)\right|Sdx$$

(2) $$q(t)=\frac{Q(t)}{Sd}=\frac{1}{d}\int_{0}^{d}\left|\rho (x,t)\right|dx$$

where *Q*(*t*) is the amount of charge accumulation within the sample, *q*(*t*) is the average charge density, *ρ*(*x*,*t*) is the space charge density, *d* is the sample thickness, *S* is the area of the electrode, and *x* and *t* are position and time, respectively. 

**Figure 7:**
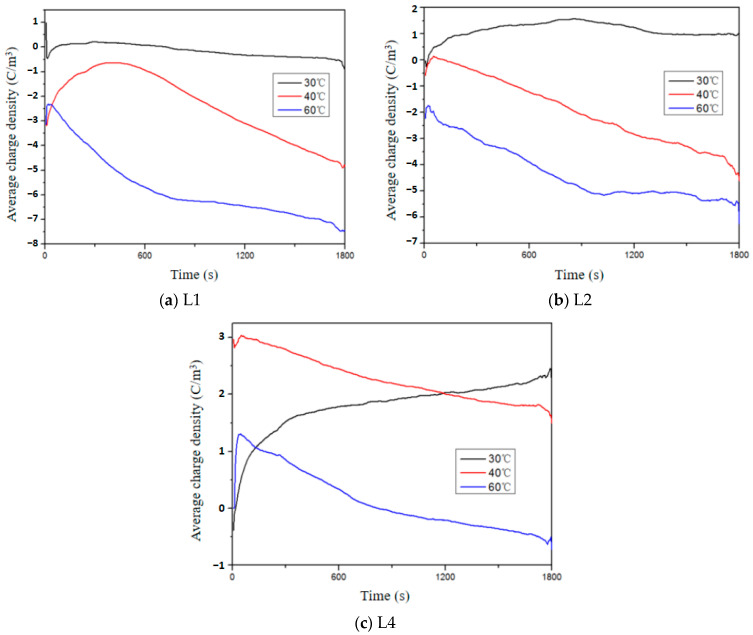
Average charge density of XLPE with different aging times during polarization at different temperatures.

Figure 7 illustrates the average charge density of the aged samples during polarization under different temperatures and an electric field strength of 80 kV/mm. It is evident that there are notable disparities in the space charge accumulation among samples with varying aging durations. Figure 7a presents the test results of a sample aged for 4 days. At 30 °C, only a marginal quantity of negative charges accumulates within the sample. At 40 °C, the absolute value of the average charge density decreases from 0 to 500 s, indicating heterocharge accumulation during the initial polarization stage due to temperature-induced recombination between heterocharges and homocharges generated by impure molecule pyrolysis reactions. At 60 °C, significant negative charge accumulation occurs within the sample. Based on the analysis in Section 3.1, it can be concluded that elevated temperature and electric field strength promote charge injection. Therefore, high-temperature-induced negative charges can be attributed to homocharge injection from electrodes. Similar phenomena are observed in samples aged for 10 days (Figure 7b). Notably, compared to samples with shorter aging durations (Figure 7c), the sample aged for 14 days exhibits increased charge accumulation at 30 °C, suggesting that thermal aging reduces the minimum temperature required for charge injection.

**Figure 8:**
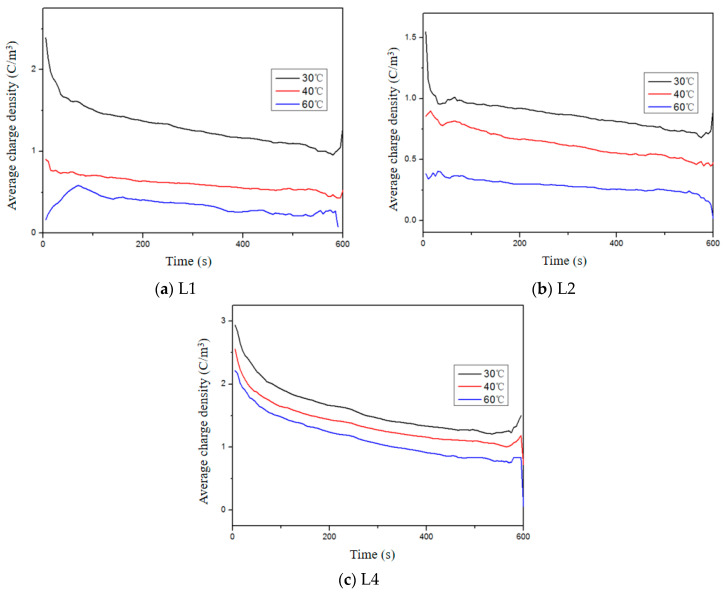
Average charge density of XLPE with different aging times during short-circuit at different temperatures.

The average charge density of aged samples during the short-circuit process under different temperatures is illustrated in Figure 8. It is evident that there are notable variations in the dissipation of space charges among different samples. With an increase in short-circuit time, the average charge density decreases rapidly, exhibiting a fast-to-slow rate of decrease. Previous studies have indicated that electron mobility surpasses hole mobility and negative charge traps tend to be relatively shallow. Consequently, at the onset of the short-circuit, a substantial quantity of negative charges promptly detraps and dissipates, leading to a rapid decline in the average charge density. Furthermore, as temperature rises, the residual space charge diminishes during the short-circuit process. This can be attributed to two factors: firstly, at higher temperatures, when polarization concludes, injected carriers from one electrode have already migrated towards the opposite electrode for recombination, thus resulting in a lower average charge density during the initial stage of the short-circuit; secondly, elevated temperatures facilitate sufficient energy acquisition by charge carriers for detrapment and dissipation. Therefore, compared to lower temperatures, higher temperatures yield a lower average charge density overall. In summary, as aging intensifies, the average charge density exhibits an initial increase at the beginning of the short-circuit, which further validates that thermal aging promotes space charge injection.

### 3.4. Electric Field Distribution Characteristics of Thermally Aged XLPE

**Figure 9:**
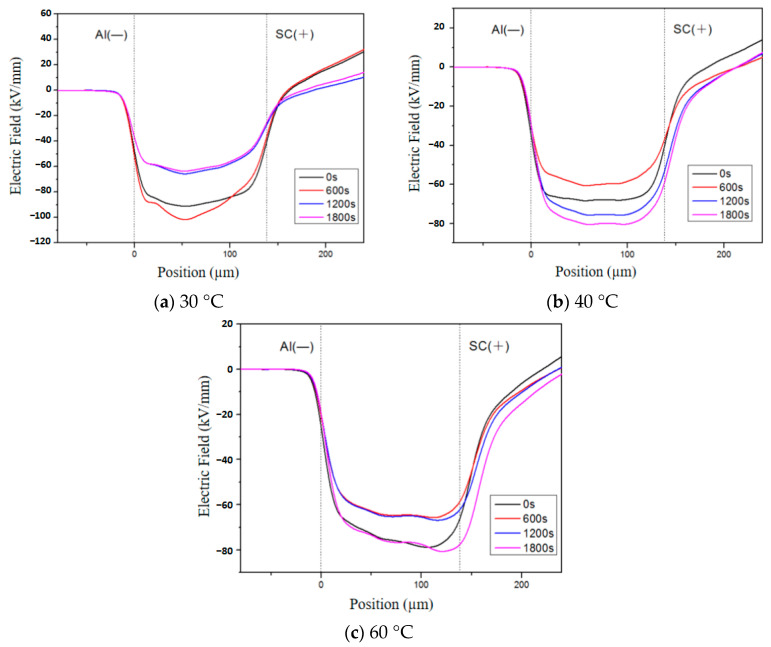
Electric field during polarization of XLPE aged for 10 days at different temperatures.

The spatial distribution of space charges in the sample will impact the electric field distribution within the sample [32]. If charges accumulate or are injected near the electrode with a polarity opposite to that of the electrode, an electric field opposing the applied electric field will be generated within the sample, thereby attenuating it [33]. Conversely, when both charge and electrode polarity are aligned, it will enhance the local electric field. Figure 9 illustrates variations in electric field distribution for samples aged at different temperatures over a period of 10 days (with an applied electric field strength of 80 kV/mm). It can be observed that as temperature increases, the electric field strength near the cathode is lower than that near the anode, indicating a higher quantity of charges being injected into the cathode compared to those injected into the anode. Furthermore, quantifying distortion in terms of the electric field distortion rate allows us to assess changes in electrical fields using the following formula:

(3) $$E_{a}=\left|\frac{E_{\max}-E_{0}}{E_{0}}\right|\times 100\%$$

where *E*_α_ is the electric field distortion rate, *E*_max_ is the maximum electric field within the sample, and *E*_0_ is the applied electric field.

The maximum rates of electric field distortion for the samples aged for 2, 10, and 16 days are measured at 6.83%, 26.94%, and 108.1% respectively. It is evident that the intensification of thermal aging amplifies the injection of homocharges, resulting in pronounced electric field distortion and a subsequent reduction in insulation lifespan.

**Figure 10:**
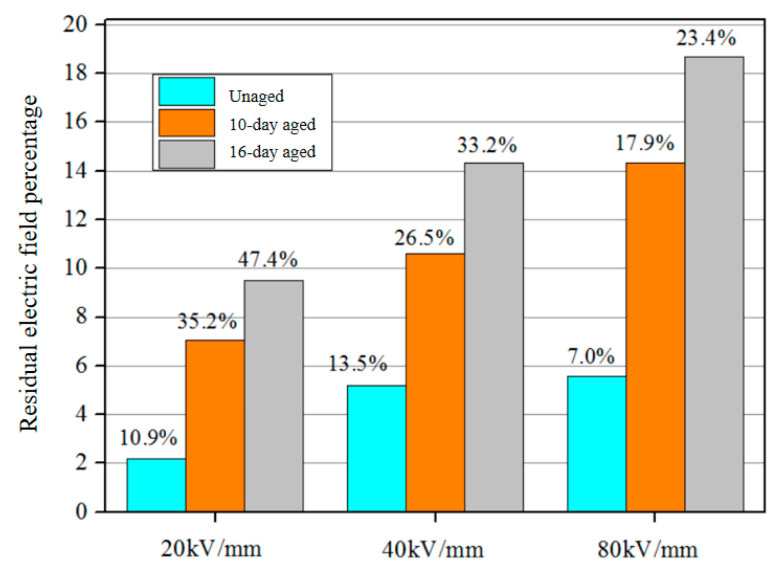
Residual electric field percentage of XLPE with different aging times and different electric fields.

The injected homocharges during polarization are captured and form space charges, leading to distortion of the electric field. During short-circuit conditions, the charges trapped by deep traps do not immediately detrap and instead create residual built-in electric fields within the sample. The percentage of residual electric fields in samples with different aging times under varying electric fields was calculated and is presented in Figure 10. It can be observed that an increase in the applied electric field results in a higher presence of residual electric fields within the sample, which can be attributed to increased injection of space charges. Furthermore, as the degree of aging intensifies, a larger residual electric field becomes evident in the sample.

### 3.5. Electrical Conduction Current Characteristics

**Figure 11:**
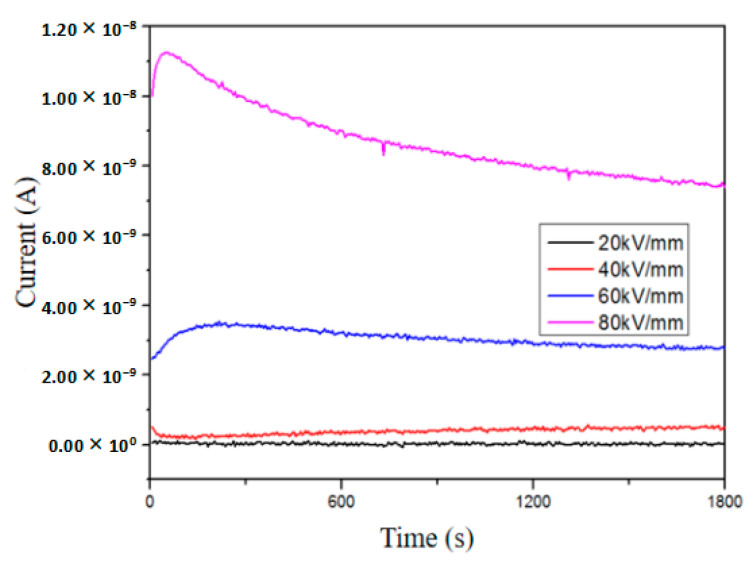
Leakage current of unaged XLPE under different electric fields.

To further elucidate the impact of aging on space charge, we analyzed the electrical conduction current of XLPE under electric fields ranging from 20 kV/mm to 80 kV/mm and temperatures between 30 °C and 60 °C. Figure 11 illustrates the leakage current of the unaged sample during polarization under different electric fields at a temperature of 30 °C. It is evident that the leakage current increases with increasing applied electric field. During the polarization process, the leakage current initially decreases before gradually stabilizing. Leakage current comprises displacement current resulting from dipole polarization and conduction current arising from carrier migration [34], with dipole polarization establishing within microseconds. Hence, the decrease in leakage current observed during initial polarization can be attributed to conduction currents. According to Ohm’s law for total currents, we can calculate the conduction current density as [35]:

(4) $$J_{c}(x,t)=J_{L}(t)-J_{d}(t)=J_{L}(t)-\varepsilon_{0}\varepsilon_{r}\frac{\partial E(x,t)}{\partial t}$$

where *J*_c_ is the conduction current density, *J*_L_ is the leakage current density, *J*_d_ is the displacement current density, *ε*_0_ is the permittivity of vacuum, and *ε*_r_ is the relative permittivity of samples.

**Figure 12:**
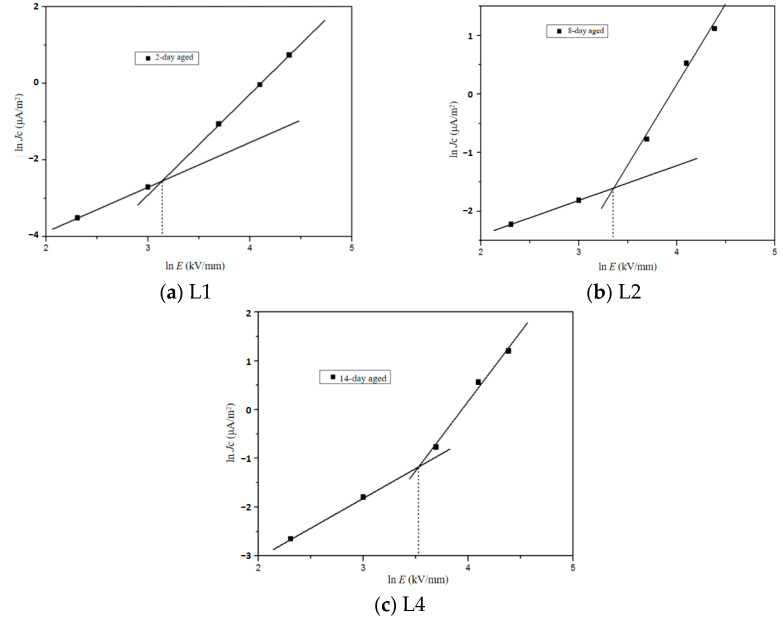
Electrical conduction current density of XLPE with different aging times under different electric fields.

The ln*J*c-In*E* characteristic curves of samples with different aging times under varying electric fields are presented in Figure 12. It is evident that the conduction current density exhibits an exponential increase with the applied electric field. Notably, the rate of increase in conduction current density is relatively slower at lower electric fields but becomes more pronounced at higher electric fields. This behavior can be attributed to a change in the mechanism governing conduction current as the applied electric field increases. The fitting results for conduction current density reveal a distinct inflection point, consistent with the theory of space charge limited current. In regions characterized by low electric fields, dielectric materials exhibit Ohmic conductivity and adhere to Ohm’s law, thus referred to as the Ohm conductivity region. The steady-state relationship between current density and electric field can be described by the following [35]:

(5) $$j=e\mu n_{0}E$$

where *e* is the electron charge, *μ* is the carrier mobility, and *n*_0_ is the carrier concentration at thermal equilibrium. When the applied electric field surpasses the space charge injection threshold *E*_Ω_, a significant increase in injected charge occurs, leading to the accumulation of a substantial amount of space charge within the sample and resulting in the formation of space charge limited current (SCLC). Consequently, this transition causes the sample to shift from the Ohmic current region to the SCLC region. The injected charges are captured and form space charges within this SCLC region. The expression for space charge limited current density can be found in reference [36] as follows:

(6) $$j_{S}=\frac{9}{8}\frac{\mu \varepsilon_{0}\varepsilon_{r}U^{2}}{d^{3}}$$

where *j*_s_ is space charge limited current density and *U* is the applied voltage. The conduction current density curve can be divided into two distinct regions: the Ohmic conduction region and the SCLC region, where a majority of the injected charges are captured.

**Figure 13:**
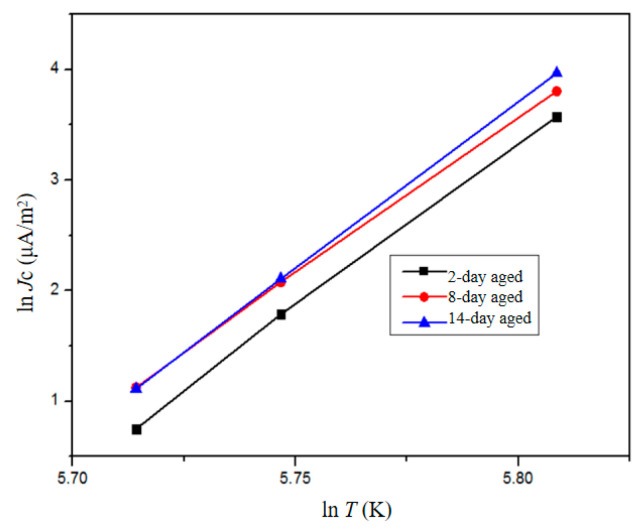
Electrical conduction current density of XLPE with different aging times under different temperatures.

The relationship between the conduction current density and temperature of aged samples is depicted in Figure 13. As the temperature rises, there is an exponential increase in the conduction current density. Furthermore, the conduction current density exhibits a correlation with conductivity, which can be expressed as follows:

(7) J=σE

where *σ* is the conductivity. Considering an electric field strength of 80 kV/mm, as per Equation (7), it is evident that the sample’s conductivity exhibits a positive correlation with temperature variations. At the microscopic level, conductivity can be mathematically represented as follows:

(8) $$\sigma =qn_{0}\mu$$

Therefore, based on Equation (8), the increase in conductivity can be attributed to the rise in carrier concentration. Furthermore, in polymers, conductivity is also influenced by carrier jump conduction. As temperature increases, carrier mobility significantly improves and charge carriers are more easily injected, leading to an enhancement in sample conductivity and an increase in conduction current density.

## 4. Conclusions

In this study, the impact of thermal aging on the space charge distribution and conduction current of XLPE cable insulation was investigated. The findings are presented below.

(1) The heterocharges in the samples originate from sample pyrolysis. The homocharges near the electrode/sample interface are influenced by the potential barrier at the interface. Following thermal aging, charges find it easier to be injected from the electrode into the sample under high electric fields and subsequently migrate within.

(2) The rise in temperature enhances the concentration of charge carriers generated by the pyrolysis reaction, thereby promoting an increase in heterocharge accumulation. As the temperature continues to elevate, the reduction in interface potential barrier facilitates the recombination of positive and negative charges, resulting in a decline in heterocharges.

(3) As thermal aging progresses, the accumulation of heterocharges diminishes, while the accumulation of homocharges amplifies. The injection of homocharges induces a pronounced distortion in the electric field near the electrode. During a short-circuit event, owing to the presence of deep traps, the rate of charge dissipation exhibits an initial rapid decline followed by a gradual decrease. Furthermore, intensified aging results in an augmentation in both deep trap density within the sample and residual electric field.

(4) As the applied electric field increases, the depth of charge injection is enhanced. Additionally, an increase in the applied electric field can also result in a rise in conduction current density. When the electric field is below the threshold value, the sample exhibits Ohmic behavior. However, as the electric field gradually surpasses this threshold, a transition to a space charge limited conduction (SCLC) region occurs.

The present study qualitatively investigated the impact of thermal aging on the spatial distribution of space charge and conduction current in XLPE cable insulation. However, a limitation lies in the absence of quantitative analysis, which necessitates further experimental exploration and can be examined in conjunction with aging models.

## Figures and Tables

**Table 1:** Sample information.

Sample Conditions	Numbers
Unaged XLPE	F
Aged for 4 days	L1
Aged for 10 days	L2
Aged for 12 days	L3
Aged for 14 days	L4

## Data Availability

Data are contained within the article.
